# Supplementary material for: ProCPU Is Expressed by (Primary) Human Monocytes and Macrophages and Expression Differs between States of Differentiation and Activation
Source: Int J Mol Sci. 2023 Feb 13;24(4):3725. doi: 10.3390/ijms24043725 (PMC9967989; doi:10.3390/ijms24043725)
Supplement: Supplementary file 1 [file ijms-24-03725-s001.zip › ijms-2126052-supplementary.pdf]

International Journal of *Molecular Sciences*

MDPI

Supplementary Materials: ProCPU is Expressed by (Primary) Human Monocytes and Macrophages and Expression Differs between States of Differentiation and Activation

Karen Claesen, Joni De Loose, Pieter Van Wielendaele, Emilie De bruyn, Yani Sim, Sofie Thys, Ingrid De Meester and Dirk Hendriks

File S1. Validation of a quantitative reverse transcriptase-polymerase chain reaction (RT-qPCR) assay to study *CPB2* mRNA expression

1. Materials and methods

1.1. Primer selection for reference genes and the target gene CPB2

A pool of twelve candidate reference genes was chosen based on their common use as endogenous reference genes in human gene expression studies. Primers were designed based on sequences from existing literature and the PrimerBank database (Massachusetts General Hospital) [1–3]. All primer pairs were designed for the primers to be located on different exons or for one of the primers to be situated on an exon-exon junction. This to ensure the specific amplification of cDNA and not genomic DNA (gDNA). In-silico specificity analysis using the primer-BLAST tool was conducted to verify the specificity of the selected primers. Primers were synthesized by Integrated DNA Technologies (IDT, USA). The sequences, length of products, predicted melting temperature (Tm) and source sequences are listed in Table S1.

A primer/probe set for the gene of interest (*CPB2*) was selected from literature (Table S2) [4,5] and purchased from IDT. The probe was labeled with FAM (6-carboxyfluorescein) as the reporter dye at the 5′-end, an internal ZEN™ quencher and with a second quencher at the 3′-end (ABkFQ). These primers and probe were also designed to minimize the signal generated from possible contaminating gDNA.

Table S1. Candidate reference genes and corresponding primer pairs evaluated in this study.

Gene		PrimerBank accession	Primer sequence forward/reverse (5′ to 3′)	Amplicon size (bp)	Predicted Tm (°C)
ARPC1a	Actin related protein 2/3 complex subunit 1A	300360514c1	ATTGCCCTCAGTCCCAAT<br>AATCACAAGTGACAATG<br>CGGTCGC	143	81.0
B2M	Beta-2-microglobulin	37704380c1	CGCTACTCTCTCTTTCTG<br>GATTTGACTTTCCATTCT<br>CTGC	111	81.0
CD71	Transferrin receptor	332309170c3	ATCGGTTGGTGCCACT<br>GAATGGACAACAGTGGGCT<br>GGCAGAAAC	131	79.0
CycA	Cyclophelin A pair 1	45439319c1	GGCAAATGCTGGAC<br>CCAACACATGCTGGTCT<br>TGCCATTCCTGGA	161	81.5
EMC7	ER membrane protein complex subunit 7	14211875a1	CTTCAGGACTGGATC<br>TCGGCGGGATCAAAT<br>CTGTAAGCTGGA	151	80.0
GUS	Glucuronidase beta	268834191c3	CTCATTTGGAATTTTGC<br>CGATCCGAGTGAAGATC<br>CCCTTTTTA	81	80.0

HPRT1	Hypoxanthine phosphoribosyl transferase	164518913c1	CCTGGCGTCGTGATTAG TGATAGACGTTCAGTCCT GTCCATAA	131	79.5
PGK-1	Phosphoglycerate kinase 1	183603937c2	GAACAAGGTTAAAGCCG AGCCGTGGCAGATTGA CTCCTACCA	137	83.5
PSMB2	Proteosome 20S subunit beta 2	315139005c1	ATCCTCGACCGATACTA CACACGAACACTGAAGG TTGGCAGAT	118	83.0
RPS8	Ribosomal protein S8	4506742c1	GCTCAGAGTGTTGTACTC GTAAAAGCACGATGCAA TTCTTCACC	106	81.0
TBP	TATA box binding protein	285026518c3	GAGCCAAGAGTGAAGA ACAGTCGCTCCCCACCAT ATTCTGAATCT	116	78.0
YWHAZ	Tyrosine 3-monoxygenase/ tryptophan 5-monoxygenase activation protein zeta	208973243c2	TGTAGGAGCCCGTAGGT CATCGTGAAGCATTGGGG ATCAAGA	179	79.0

Bp: base pairs; conc: concentration of forward and reverse primer used for the validation of the primer pair; Tm: melting temperature.

Table S2. Sequences of the primers and probe for detection of the *CPB2* gene by RT-qPCR.

Gene		Primer	Primer sequence (5′ to 3′)	Amplicon size (bp)	Conc (nM)
CPB2	Carboxypeptidase B2	Forward	TGCATCGGAACAGACCTGAA	65	500
		Reverse	CTGGATGCACCTTCCTCACA		500
		Probe	6FAM-TTGCTTCCA-ZEN-AACACTG-AbkFQ		200

6FAM: 6-carboxyfluorescein (5′-end reporter dye); AbkFQ: nonfluorescent quencher (3′-end nonfluorescent quencher); bp: base pairs; conc: concentration of forward and reverse primer used for validation of the primerpair; RT-qPCR: quantitative reverse transcriptase-polymerase chain reaction: ZEN: internal quencher.

1.2. Quantitative reverse transcriptase-polymerase chain reaction (RT-qPCR) analysis

RT-PCR amplification reactions were performed using the CFX Connect™ Real-Time PCR System (Bio-Rad, USA). The reaction mixture (20 µL) contained 10 µL 2X SSoAdvanced™ Universal SYBR® Green supermix (Bio-Rad, USA), 1 µL (250 nM, 300 nM or 500 nM depending on the primer set; Table S3) of each primer, 5 µL cDNA and 3 µL nuclease-free water as recommended in the manufacturer’s instructions. For amplification of CPB2, the 2X SSoAdvanced™ Universal SYBR® Green supermix and 2 µL nuclease-free water were substituted by an equal volume of 2X SSoAdvanced™ Universal probes supermix (Bio-Rad, USA) and probe (200 nM; IDT, USA). All samples were run in triplicate and each run included a no-template control (nuclease-free water instead of sample) for the genes included in the run. Thermocycling conditions used were as follows: one cycle of 95 °C for 30 s, followed by 40 cycles of denaturation at 96 °C for 15 s and annealing and extension with fluorescent plate read at 60 °C for 40 s. Afterward, a melt curve was obtained by melting the amplicon from 60 to 95 °C to check for primer dimers. Data, including individual quantification cycle (Cq) values for each gene, were acquired using the CFX Manager software.

Table S3. Candidate reference genes and corresponding primer pairs evaluated in this study.

Gene	Primer concentration	Range of Cq values	Efficiency (%)	Slope	Y-intercept	R^2
ARPC1a	250 nM	27.19 – 32.85	91.2	-3.6	44.5	0.999
B2M	250 nM	19.57 – 28.59	109.0	-3.1	40.2	0.991
CD71	200 nM	22.60 – 32.04	98.3	-3.4	42.9	0.996
CycA	200 nM	24.26 – 33.48	93.3	-3.5	38.9	0.998
EMC7	250 nM	29.16 – 36.65	92.1	-3.5	43.9	0.998
GUS	250 nM	29.10 – 36.85	102.2	-3.3	42.0	0.996
HPRT1	250 nM	23.13 – 33.17	134.7	-2.7	40.0	0.931
PGK-1	250 nM	19.51 – 30.16	131.2	-2.7	36.5	0.972
PSMB2	250 nM	27.52 – 33.15	97.4	-3.4	40.7	0.994
RPS8	250 nM	23.56 – 33.65	95.9	-3.4	37.0	0.995
TBP	200 nM	27.18 – 33.99	98.5	-3.4	43.5	0.996
YWHAZ	250 nM	19.26 – 32.07	103.0	-3.3	39.1	0.993
CPB2	500 nM	19.19 – 30.75	100.1	-3.3	39.1	0.999

Cq: quantification cycle.

1.3. Primer specificity and PCR amplification efficiency of the selected primer pairs

To validate that accurate template quantification is achieved with the selected primer pairs, primer specificity (verification that each reaction amplifies the correct target) and PCR amplification efficiency were determined. The specificity of each primer pair was evaluated by melt curve analysis, agarose gel electrophoresis on the RT-qPCR products and sequencing of purified RT-qPCR products. For agarose gel electrophoresis, PCR products were loaded onto a 2% agarose gel with TBE buffer, ran for 75 min at 100 V (Mini Sub-Cell GT Cell and PowerPac Basic Power Supply, Bio-Rad, USA) and stained with Gel Red (Sigma-Aldrich, USA). Visualization was performed on an OptiGo Gel Imaging System (Isogen Life Sciences, The Netherlands) with UV-light. The amplification efficiency of each primer pair was determined using a relative standard curve constructed by performing RT-qPCR on each gene using a 5-fold serial dilution series of template cDNA from HepG2 cells. Individual Cq values were plotted against the logarithm of the dilution factor whereafter PCR amplification efficiencies (E) were calculated from the slope of the respective relative standard curve by the formula $E(\%) = 10^{-1/slope} -1*100$ and R^2 values were obtained from the lines of best fit.

1.4. Gene stability analysis and reference gene selection

To accurately perform relative quantification, normalization of the gene expression is required to correct for non-specific experimental variations [6]. Therefore, the expression stability of the candidate reference genes in the different cell types (HepG2, THP-1, PMA-stimulated THP-1 cells, primary human monocytes, primary human M-CSF macrophages, primary human IFN-γ/LPS-stimulated [M1]- and IL-4-stimulated [M2] macrophages) was evaluated by RT-qPCR. Cq values for all samples were calculated. The stability of the reference genes was determined, the optimal combination of reference genes for normalization was selected and normalization factors (NFs) of the selected reference genes were calculated using the geNorm algorithm of the qbase+ software (Biogazelle, Ghent, Belgium) [6,7].

2. Results

2.1. Primer specificity, efficiency and expression profiling of candidate reference genes

A total of twelve candidate reference genes were targeted to select suitable internal controls for gene expression studies using RT-qPCR. The specificity of the primer pairs was confirmed via agarose gel electrophoresis, melt curve analysis and Sanger sequencing.

Single sharp bands at the expected MW were present on the agarose gel for the amplicons of all reference genes, except for PSMB2, RPS8 and PGK-1 (Figure S1). For the latter, a diminished band appeared on the gel underneath the main band. This is suspicious for the presence of various DNA sequences (e.g. primer dimers or non-specific

amplification products). Therefore, the specificity of these three reference genes was analyzed more closely using Sanger sequencing and melt curve analysis.

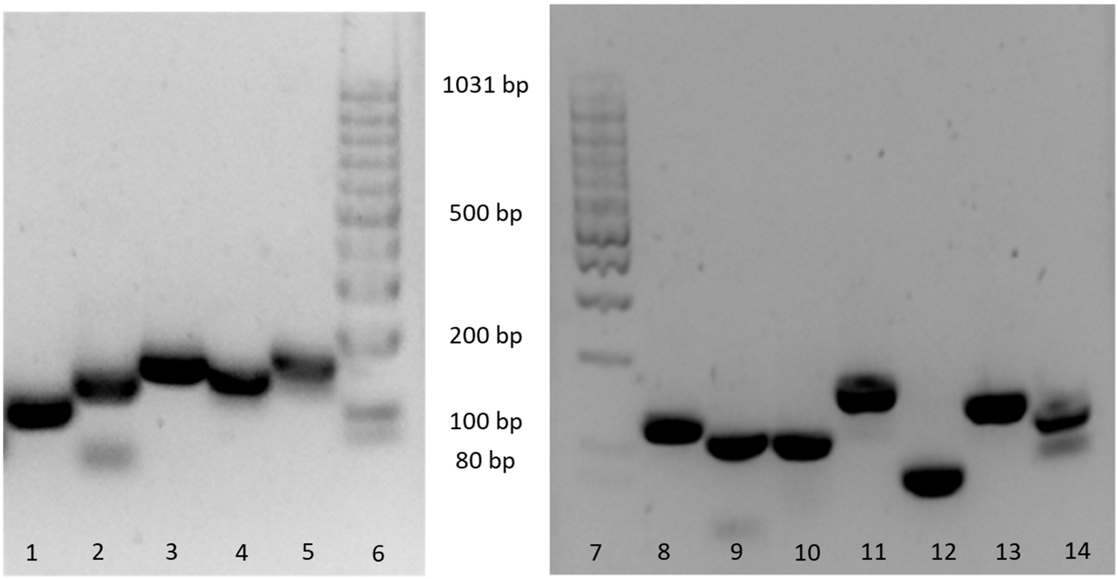

Figure S1. Specificity of primers. Agarose gel electrophoresis showing amplification specificity of the candidate reference genes. Lane 1: B2M; lane 2: PSMB2; lane 3: ARCP1a; lane 4: CD71; lane 5: CycA; lane 6-7: low range DNA ladder; lane 8: HPRT1; lane 9: RPS8; lane 10: TBP; lane 11: YWHAZ; lane 12: GUS; lane 13: EMC7; lane 14: PGK-1. Bp: base pairs.

Sanger sequencing is a PCR-based sequencing method that allows to unambiguously verify the identity of the amplified PCR products by determining the actual nucleotide sequence of the fragments after purification from the qPCR reactions. Sequencing of the primer pairs PSMB2, RPS8 and PGK-1 was successful with a pairwise identity between 96.9-100.0%, confirming amplification of the correct sequence (data not shown). In addition, the melt curves of these three genes, and also those of the other candidate reference genes, showed a single peak. The presence of this single peak in the melt curve indicates that the primers amplified specific products (Figure S2).

Next, the amplification efficiency of the primers was determined and ranged from 91.2 to 134.7%. R^2 was > 0.93 for all primers (Table S3). Only those primer pairs with an amplification efficiency between 90 – 110 % and an R^2 > 0.98 were retained as candidate reference genes. As a result HPRT1 (efficiency = 134.7% and R2 = 0.931) and PGK-1 (efficiency = 131.2% and R^2 = 0.97) were henceforth excluded as potential reference genes.

The Cq values obtained by RT-qPCR were used to provide an overview of the expression levels of the candidate reference genes across all the samples. The mean Cq values of all primer pairs for the different samples ranged from 19.57 to 36.85, which is acceptable for reliable RT-qPCR quantification. ARPC1a showed the least variation in its transcript level with a coefficient of variation (CV) of 7.3%, across all samples and TBP showed the second least variation in gene expression with a CV of 7.8%, followed by EMC7 (CV = 8.69%). B2M was the most variable reference gene (CV = 18.4%) followed by RPS8 (CV = 17.7%) (Figure S3).

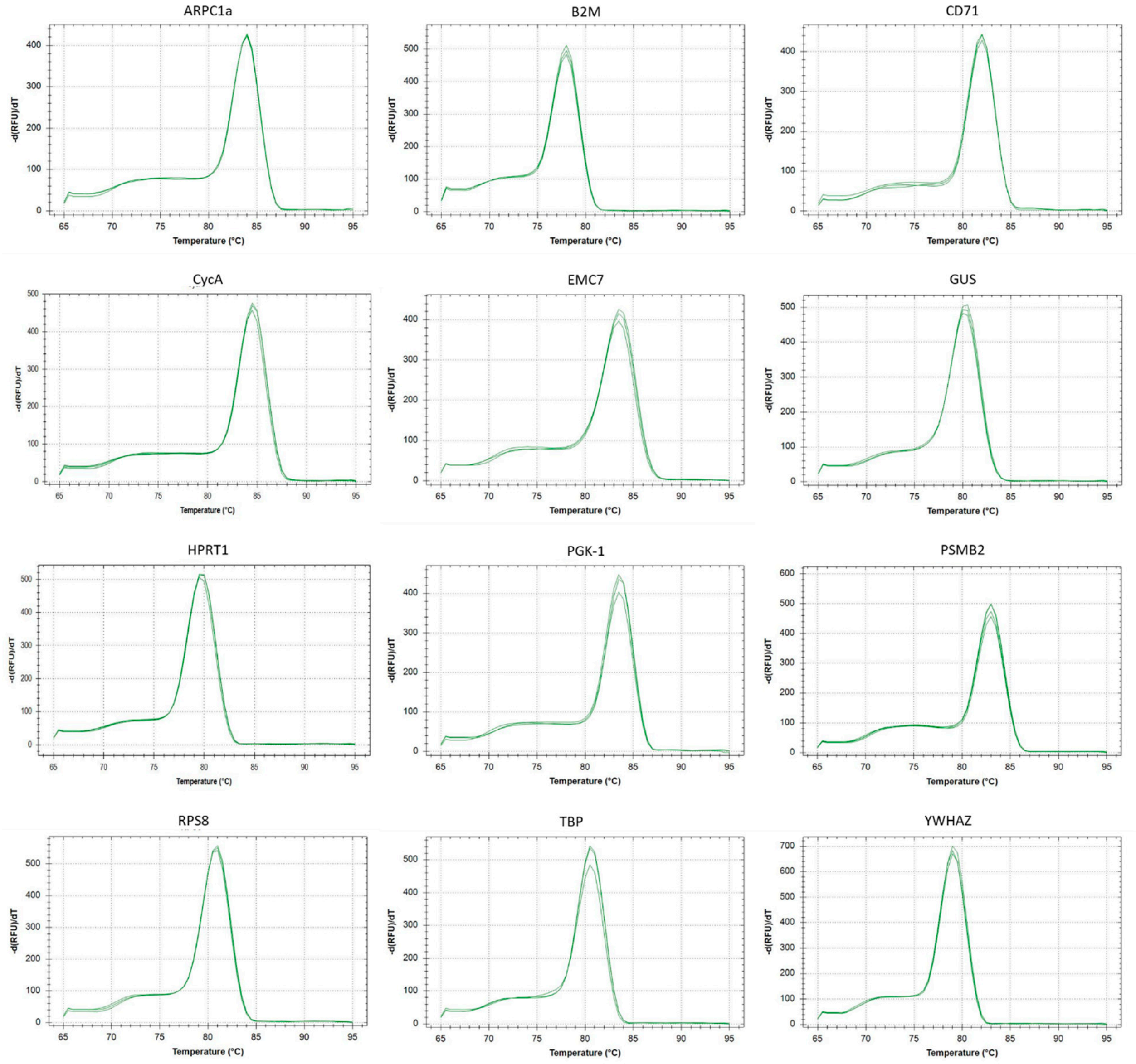

Figure S2. Specificity of RT-qPCR amplification. Melt curves of the 12 candidate reference genes with single peak after RT-qPCR reactions. RFU: relative fluorescence units, RT-qPCR: quantitative reverse transcriptase-polymerase chain reaction.

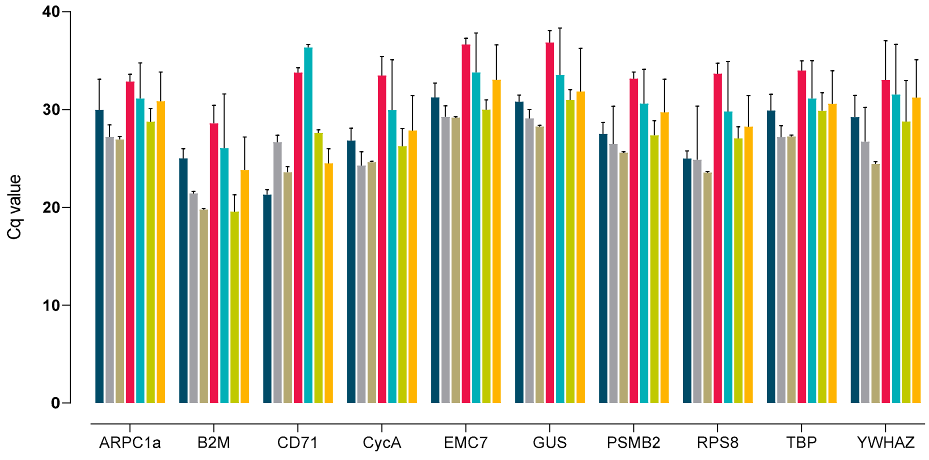

Figure S3. Expression profiles of candidate reference genes across all the experimental samples. The expression profiles of 12 candidate reference genes in absolute quantification cycle (Cq) values over different samples including from left to right HepG2 (dark blue), THP-1 (grey), PMA-stimulated THP-1 cells (brown), primary human monocytes pink), primary human M-CSF macrophages (light blue), primary human IFN-γ/LPS stimulated macrophages (green) and primary human IL-4-stimulated macrophages (yellow).

2.2. Analysis of expression stability and determination of the optimal number of reference genes

Using qbase+ software, the mean expression stability of the candidate reference genes was defined by allocating a so-called M-value to each gene in a pool of potential internal control genes. An M-value is the mean pairwise variation between an individual gene and the other putative reference genes tested [6,7]. Genes with the lowest M-values have the most stable expression. GeNorm analysis revealed that the most stably expressed gene was TBP with an M-value of 0.465, followed by EMC7 (M = 0.505) and ARPC1a (M = 0.550). B2M was the least stable gene for RT-qPCR normalization (Figure S4A).

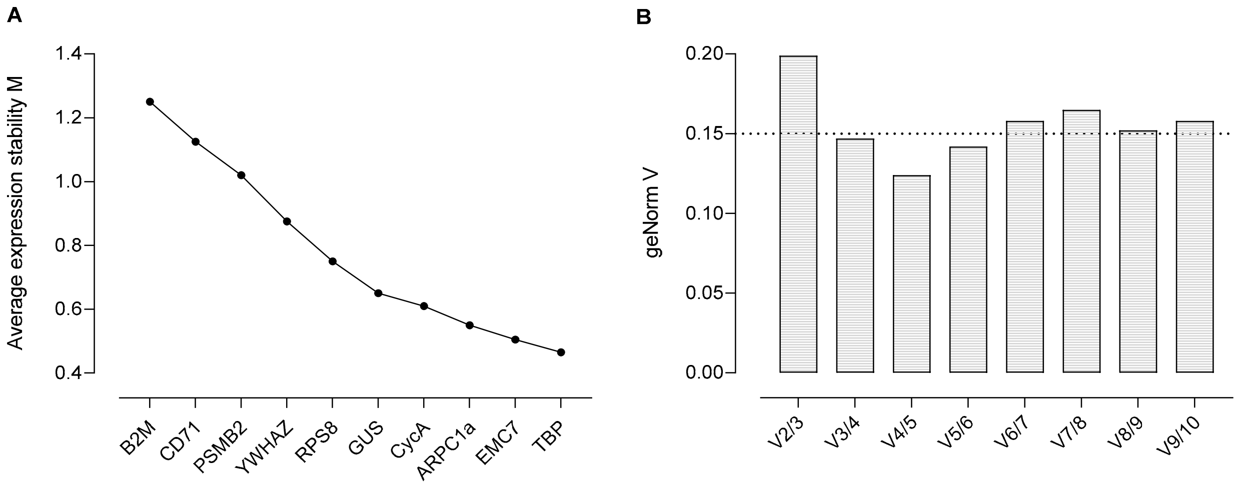

Figure S4. A) Average expression stability values of remaining potential internal control genes. Average expression stability values (M) during stepwise exclusion of the least stable internal control genes by geNorm application. The highest M values corresponded to the least stable gene, B2M; while the lowest M values corresponded to the most stable gene, TBP. **B) Determination of the optimal number of internal genes for accurate normalization**. The pairwise variation (V = Vn/Vn+1) was calculated by geNorm to determine the optimal number of reference genes required for RT-qPCR normalization. Values under 0.15 indicate that no additional genes are required for the normalization. Here the optimal number of reference genes is three.

The optimal number of reference genes required for accurate normalization to obtain precise RT-qPCR results was also determined by pairwise variation (Vn/Vn+1) using geNorm [8]. According to Vandesompele *et al.*, a cutoff value of Vn/Vn+1 < 0.15 suggests that the addition of another reference gene would have no significant contribution to normalization in RT-qPCR analysis [6,7]. As shown in Figures S4B, the V2/3 pairwise variation (0.199) was higher than

0.15, while the V3/4 variation was 0.147. Therefore, the combination of three genes (ARPC1a, EMC7 and TBP) is good enough to perform accurate RT-qPCR normalization.

File S2. Appropriate stimulation of primary M-CSF macrophages was confirmed by ELISA

Appropriate macrophage activation was confirmed beforehand by measuring the concentration of different cytokines (TNFα, IL-6, IL-1β and IL-10) in the supernatant of unstimulated and stimulated macrophages by use of ELISA and comparing the concentration of these cytokines in all groups. Stimulation of primary M-CSF primed macrophages with a combination of IFN-ɣ and LPS resulted in a significant increase in pro-inflammatory TNFα, IL-6 and IL-1β secretion (Figure S5). A clear decrease in pro-inflammatory cytokine production (TNFα, IL-6 and IL-1β) and a substantial increase in the concentration of the anti-inflammatory cytokine IL-10 confirmed successful differentiation of primary M-CSF primed macrophages into an anti-inflammatory subtype after IL-4-stimulation.

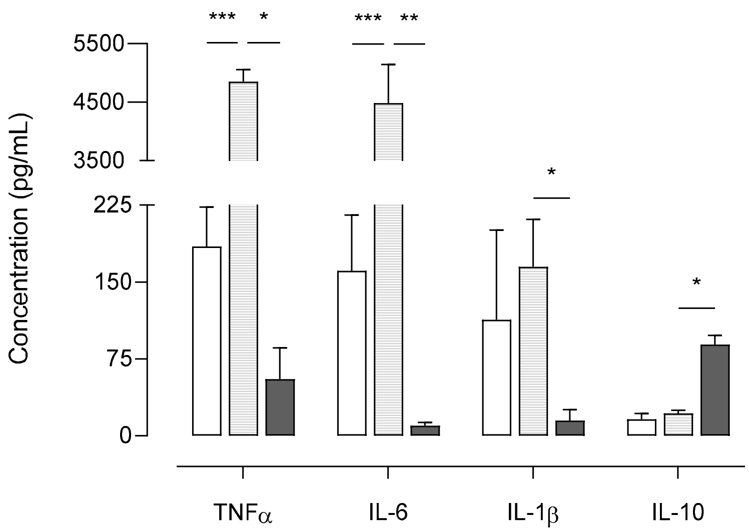

Figure S5. Appropriate stimulation of primary human M-CSF macrophages with IFN-γ/LPS or IL-4 was confirmed by ELISA. TNFα (8h after activation), IL-6, IL-1β and IL-10 (all 24h after activation) levels were determined in the medium of primary human M-CSF primed macrophages (white), primary human IFN-γ/LPS-stimulated macrophages (light grey) and primary human IL-4-stimulated macrophages (dark grey). Results are presented as mean ± SEM (N = 4 – 6). p-values were calculated by performing a Kruskal-Wallis test with Dunn's multiple comparison test; * $p < 0.05$, ** $p < 0.01$ and *** $p < 0.001$.

File S3. Immunofluorescent staining shows proCPU/CPU inside (primary) human monocytes and macrophages

1. Materials and methods

HepG2, THP-1 and CD14+ cells were seeded in Nunc™ Lab-Tek™ II CC2™ Chamber Slides at a density of 0.25 – $1x10^5$ cells per well (depending on the cell type) and incubated at 37 °C under 95% air/5% CO_2 atmosphere. Cell culturing and differentiation protocols were identical as described. At the end of the respective culturing protocol, the cells were washed with HBSS and fixed with 4% paraformaldehyde (PFA) for 30 min at room temperature. Following a washing step with PBS cells were permeabilised with 0.1% Triton X-100 in blocking buffer (2% bovine serum albumin, 5% normal goat serum in PBS) for 10 min, followed by a subsequent blocking step in blocking buffer for 30 min at room temperature. Next, the cells were incubated overnight at 4 °C with primary antibodies against proCPU/CPU (CP17, Agrisera; 1:100) diluted in blocking buffer. After washing in DPBS, the cells were incubated with FITC-labelled goat anti-rabbit IgG (554020, BD Biosciences; 1:200 in blocking buffer) for 1 h at room temperature protected from light. The slides were covered with a cover glass using Vectashield antifade mounting medium containing DAPI (Vector Laboratories) and visualised on an inverted Leica TCS SP8 confocal laser scanning microscope (Leica-microsystems, Wetzlar) using a HC PL APO CS2 63X/1.20 water lens. Blue fluorescence was obtained with a 405 nm diode laser and HyD detector. A white light laser (excitation wavelength of 488 nm) and a HyD detector was used to visualize FITC. The staining was checked for autofluorescence and non-specific binding of the secondary antibody. Normal rabbit IgG (Invitrogen) was used as isotype control.

2. Results

Since we did not succeed to detect proCPU/CPU protein in the cellular lysates of any of the cell types, not even in the lysate of HepG2 cells, we tried to investigate the presence of proCPU/CPU inside the different cell types by immunocytochemistry. Immunofluorescent staining with a polyclonal rabbit anti-human proCPU/CPU antibody confirmed the presence of proCPU/CPU in all cell types (Figure 5). In general, proCPU/CPU staining was diffuse and distributed throughout the cytoplasm with no granular intracellular staining pattern. All staining controls were negative (Figure 5H-J).

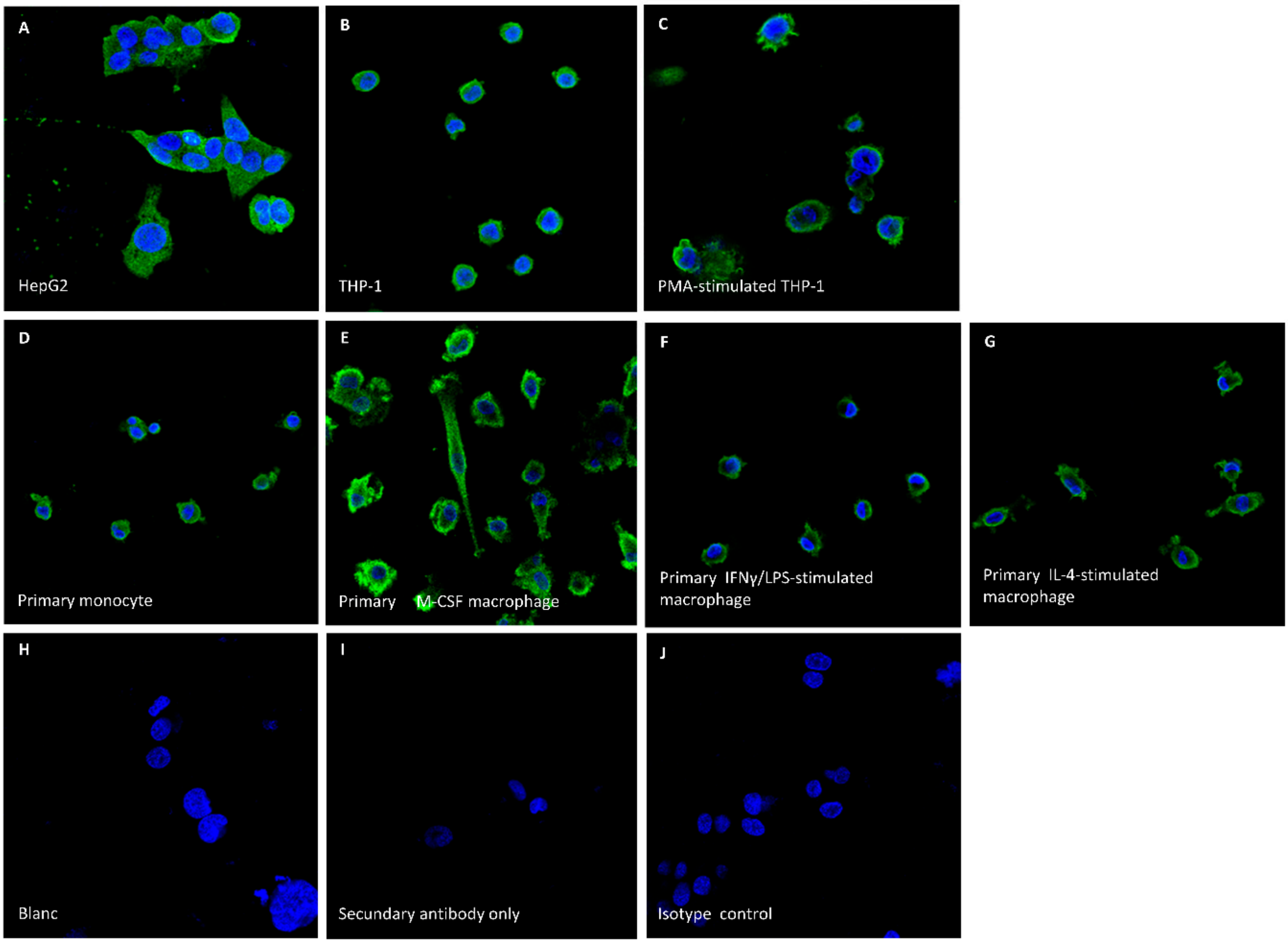

Figure S6. Staining of proCPU/CPU in HepG2 (A), THP-1 (B), PMA-stimulated THP-1 cells (C), primary human monocytes (D), primary human M-CSF macrophages (E), primary human IFN-ɣ/LPS-stimulated macrophages (F) and primary human IL-4-stimulated macrophages (G). Cells were fixed with 4% PFA, permeabilised with 0.1% Triton X-100 and stained for proCPU/CPU (green; CP17, polyclonal rabbit anti-human proCPU/CPU antibody) and DAPI (nuclear marker, blue). Staining controls (H: blanc; I: secondary antibody only; J: isotype control: normal rabbit IgG [Invitrogen, USA]) were all negative. Representative images of three independent experiments.

References

1. Spandidos A, Wang X, Wang H, Seed B. PrimerBank: a resource of human and mouse PCR primer pairs for gene expression detection and quantification. Nucl Acid Res 2010; 38: 792–9.
2. Spandidos A, Wang X, Wang H, Dragnev S, Thurber T, Seed B. A comprehensive collection of experimentally validated primers for Polymerase Chain Reaction quantitation of murine transcript abundance. BMC Genomics 2008; 9: 633.
3. Wang X, Seed B. A PCR primer bank for quantitative gene expression analysis. Nucl Acids Res 2003; 31: 1–8.
4. Lin JHH, Garand M, Zagorac B, Schadinger SL, Scipione C, Koschinsky ML, Boffa MB. Identification of human thrombin-activatable fibrinolysis inhibitor in vascular and inflammatory cells. Thromb Haemost 2011; 105: 999–1009.
5. Maret D, Boffa MB, Brien DF, Nesheim ME, Koschinsky ML. Role of mRNA transcript stability in modulation of expression of the gene encoding thrombin activable fibrinolysis inhibitor. J Thromb Haemost 2004; 2: 1969–79.
6. Hellemans J, Mortier G, De Paepe A, Speleman F, Vandesompele J. qBase relative quantification framework and software for management and automated analysis of real-time quantitative PCR data. Genome Biol 2008; 8.
7. Hellemans J, Vandesompele J. Selection of reliable reference genes for RT-qPCR analysis. Methods Mol Biol 2014; 1160: 19–26.
8. Hossain MS, Ahmed R, Haque MS, Alam MM, Islam MS. Identification and validation of reference genes for real-time quantitative RT-PCR analysis in jute. BMC Mol Biol 2019; 20: 1–13.
